# Current Concepts on the Application, Pharmacokinetics and Complications of Antibiotic-Loaded Cement Spacers in the Treatment of Prosthetic Joint Infections

**DOI:** 10.7759/cureus.20968

**Published:** 2022-01-05

**Authors:** Panagiotis V Samelis, Eftychios Papagrigorakis, Eleni Sameli, Andreas Mavrogenis, Olga Savvidou, Panagiotis Koulouvaris

**Affiliations:** 1 Orthopaedics, Orthopaedic Research and Education Center, Attikon University Hospital, Athens, GRC; 2 Orthopaedics, Children's General Hospital Panagiotis & Aglaia Kyriakou, Athens, GRC; 3 Operation Center, National Public Health Organization, Athens, GRC; 4 Orthopaedics, School of Medicine, National and Kapodistrian University of Athens, Athens, GRC; 5 Orthopaedics, Attikon University Hospital, Athens, GRC; 6 Orthopaedic Surgery, School of Medicine, National and Kapodistrian University of Athens, Athens, GRC

**Keywords:** pji, impregnation, stage, revision, pmma, antibiotic, spacer, infection, joint, prosthetic

## Abstract

Prosthetic joint infection (PJI) is a devastating complication of total joint replacement surgery. It affects about 2% of primary total joint replacements. Treatment aims at infection eradication and restoration of patient's mobility. Two-stage revision arthroplasty with an interim application of an antibiotic-loaded cement spacer (ALCS) is the widely accepted treatment for PJI. Spacers are powerful local carriers of antibiotics at the site of infection, effective against biofilm-protected microbes. On the other hand, spacers permit some mobility of the patient and facilitate final prosthesis implantation. ALCS's are either commercially available or prepared intraoperatively on prefabricated or improvised molds. Antibiotic elution from the spacer depends on the amount of the antibiotic used for cement impregnation, at the expense of mechanical stiffness of the spacer. The antibiotic should not exceed 4g per 40g of bone cement to preserve the mechanical properties of the cement. Spacers are frequently accompanied by local or systemic complications. The spacer may break, dislocate and compress vessels or nerves of the limb. Systemic complications are the result of excess elution of antibiotic and include nephrotoxicity, hepatotoxicity, ototoxicity, allergic reactions or neutropenia. Elderly patients with comorbidities are at risk to present such complications. Microbial resistance is a potential risk of long-lasting spacer retention. Persisting infection may require multiple spacer replacements.

## Introduction and background

Prosthetic joint infection (PJI) is a major complication after total joint replacement surgery [1-3]. In the United States, PJI complicates 0.3-2% of total hip replacements (THR) and 2.4% of total knee replacements (TKR) per year [2,4]. PJI is the main cause of total knee revision surgery [1] and the third cause (after aseptic loosening and dislocation) of total hip revision surgery [1,3]. A mortality rate of 8-25% per year after PJI has been reported [3].

PJI management is not easy, since there is no consensus among specialists in relation to PJI definition, diagnosis, the causative microorganism and the appropriate treatment [2,3,5]. Depending on the chronicity of the infection and the stability of the prosthesis, the treatment options for PJI are surgical debridement with prosthesis retention (debridement, antibiotics, implant retention - DAIR), surgical debridement and one stage prosthesis replacement, or surgical debridement and two-stage prosthesis replacement [1,2]. Not infrequently, poor bone or soft tissue quality of the infected joint and comorbidities of the patient do not allow any of these interventions. Thus, other treatment options may be decided, such as resection arthroplasty, arthrodesis, amputation or chronic antimicrobial chemoprophylaxis without surgery [1,2,6].

Two-stage revision arthroplasty is the widely accepted treatment for delayed and late PJI [1-3, 6-10]. The first stage involves removal of the infected prosthesis and surgical debridement of the contaminated, necrotic and granular tissue of the joint and the adjacent metaphysis and diaphysis [1]. A temporary poly-methyl-methacrylate (PMMA, bone cement) implant, impregnated with high doses of antibiotic, known as antibiotic-loaded cement spacer (ALCS), fills the gap left after debridement [1-3,5,11].

Systemic administration of antibiotics against isolated or expected microorganisms follows for the next 2-12 weeks. Elimination of the clinical and laboratory signs of infection signals transition to second stage surgery, when the final prosthesis will be implanted [1-3,11]. Antibiotic treatment is discontinued 2-8 weeks before the second stage surgery (a period termed drug holiday), to ensure eradication of the pathogen [1,2,10,12]. The spacer is removed, meticulous surgical debridement is performed and cultures are ordered (spacer sonication, tissue cultures). Subsequently, the final prosthesis is implanted [1,6,12] and fixed with cement impregnated with prophylactic dose of antibiotic [2,11]. Following second stage implantation, the patient may, or may not, receive a short-term (up to six weeks) prophylactic antibiotic regimen, based on cultures obtained during the 1st and the 2nd stage procedure [1,2,10].

## Review

History of ALCS application

In the past, PJI was an unsolved complication of primary joint replacement, leading in most cases to resection arthroplasty (Girdlestone) [13].

In 1970, Hessert and Ruckdeschel [13] and Buchholz and Engelbrecht [14], independently studied antibiotic release from antibiotic-impregnated bone cement and pointed out its potential prophylactic use for primary prosthesis fixation to reduce the risk of PJI. However, one year earlier (1969), Buchholz et al. already used antibiotic-impregnated PMMA for implant fixation either for prophylaxis (primary total hip arthroplasty) or for infection control after revision arthroplasty for PJI. For the latter procedure, Buchholz used the term “exchange arthroplasty” to describe, actually, the one-stage revision arthroplasty [14].

In 1978, Carlsson [15] and, later (1983) Rand [16] and Insall [7] described the two-stage revision arthroplasty for PJI, however, without application of antibiotic-loaded cement between stages. Prolonged immobilization of the patient (bed rest) or the limb (using a splint or an external fixator) followed first-stage debridement [7]. The final prosthesis was inserted 2-6 weeks later [7,16].

In 1979, Hovelius and Josefsson, taking into consideration of precedent experience on the management of chronic osteomyelitis with local application of antibiotic-loaded cement, described for the first time, in a case report, the two-stage replacement for hip PJI with insertion of gentamycin-infused cement spheres after first-stage prosthesis removal and debridement. The limb was immobilized by traction for three weeks, after which, the final prosthesis was implanted [17].

Nevertheless, one-stage revision frequently fails to eradicate the infection, and two-stage revision implies prolonged bed rest, joint immobilization and a challenging second-stage surgery with poor functional results due to soft tissue contraction and muscle atrophy [6,18].

In 1987, Borden and Gearen commented on the role of antibiotic-loaded beads in two-stage revision for knee PJI, not only as antibiotic carriers, but also as a temporary implant to maintain the length of the extensor mechanism of the knee [19]:

«.. the beads served the additional purpose of aiding in the maintenance of the size of the suprapatellar space and the length of the quadriceps mechanism» [19].

In 1988, Cohen et al. [20] used antibiotic-impregnated cement blocks - spacers - to fill the space created after debridement in two-stage revision of knee PJI. These ALCS’s were static, which means, they allowed some mobility of the patient but not of the joint [20]. Practically, the static spacers worked as a temporary arthrodesis between the joints. Unfortunately, prolonged immobilization of the joint from static spacers caused serious complications during the second-stage prosthesis insertion, due to soft tissue contractures, muscle atrophy and bone loss [18,21].

This problem was addressed with the introduction of articulating spacers [21]. Articulating spacers improve joint mobility between stages of replacement, leading to better conditions for second-stage surgery [6].

The role of antibiotic-loaded cement spacers in the treatment of PJI

The role of ALCS’s in the treatment of PJI is twofold: mechanical and pharmaceutical [1,2]. The ALCS fills the gap, which occurs after the removal of the infected prosthesis and surgical debridement. Thus, the ALCS prevents extensive scarring, preserves the quality of the bone and the length of the limb and facilitates final prosthesis implantation [1,6,19,20]. Before the introduction of ALCS in two-stage revision arthroplasty for PJI, prolonged immobilization of the patient and the limb between the two stages resulted in muscle atrophy, soft tissue contraction, a short limb and joint stiffness [1,7,17,19]. Moreover, technically, the second-stage surgery was extremely difficult [1,6,22].

Nevertheless, the most important role of the spacer in the management of PJI is its role as a powerful local antibiotic carrier. Systemic administration of antibiotics (iv, pos) after the first-stage surgery is effective mainly against microorganisms, which are in a free form (not in a biofilm, planktonic) [11,23]. However, in delayed or in late PJI the biofilm is well-formed [23]. Management of microorganisms, which are protected inside the biofilm, requires high local concentrations of antibiotics. To obtain such concentrations after systemic administration of antibiotics would lead to toxic levels of the antibiotic in the systemic circulation [1,11].

The ALCS is the only way to reach such high local concentrations of the antibiotic, without a concurrent increase of its concentrations in the plasma or urine [2,6,9,11]. With the ALCS, the concentration of the antibiotic reached at the site of infection is way higher (up to 700 times) than the one reached by systemic administration [1,24]. At the same time, complications that arise from high systemic doses are avoided [1]. On the other hand, the antibiotics provided by the spacer are diffused locally in tissues with decreased blood supply, where it would be impossible to reach the Minimum Inhibitory Concentration (MIC) of the causative microorganism through systemic administration [1].

The management of PJI with the use of ALCS leads to the eradication of the infection in more than 90% of cases [1,2,25]. In addition, this method leads to shorter hospitalization, less cost, increased mobility and function of the joints, less pain and improved patients' satisfaction [1,2].

Types of spacers in two-stage revision for PJI

Depending on the ability to keep joint mobility after the first-stage surgery, spacers are classified as static (non-articulating, block spacers) and articulating spacers [1,2,6].

Static Spacers

Static or non-articulating spacers consist of a concrete block of antibiotic-impregnated cement, which fills the articular gap after the first revision stage [1,19,20]. Static spacers are molded intraoperatively. They are not routinely used today, as they restrict the range of motion of the knee between the two revision stages. Thus, first-stage implantation of a static spacer results in a challenging second-stage final prosthesis insertion, with poorer long-term outcomes, due to muscle atrophy, quadriceps contracture and overall increased rigidity around the knee joint [1,2,6,21].

Articulating Spacers

Articulating spacers allow joint motion and facilitate patient ambulation during two-stage replacement. Different types of articulating spacers for hip and knee have been described, depending on the construction method (Table 1).

**Table 1:** Types of articulating antibiotic-loaded cement spacers

Types of cement spacers
Hand-made spacers: these spacers mimic either joint anatomy [26] or joint kinematics (ball and socket spacer) [9,27].
Intraoperatively constructed spacers: based on commercially available molds [28-30] or using molds, which have been created on removed and sterilized implants [6,31,32]. These spacers have good congruency and the antibiotic concentration achieved is potentially higher than that of commercial spacers.
Commercial (prefabricated), ready-to-use cement spacers [9].
Spacers with additional metal or plastic articulating elements incorporated within the cement (prosthesis with antibiotic-loaded acrylic cement, PROSTALAC) [6,22,33,34].
Application of a new or reused (autoclaving) sterilized femoral component with antibiotic-loaded cement. Stabilization of a new tibial PE insert with antibiotic-loaded cement [6,18].

**Figure 1:**
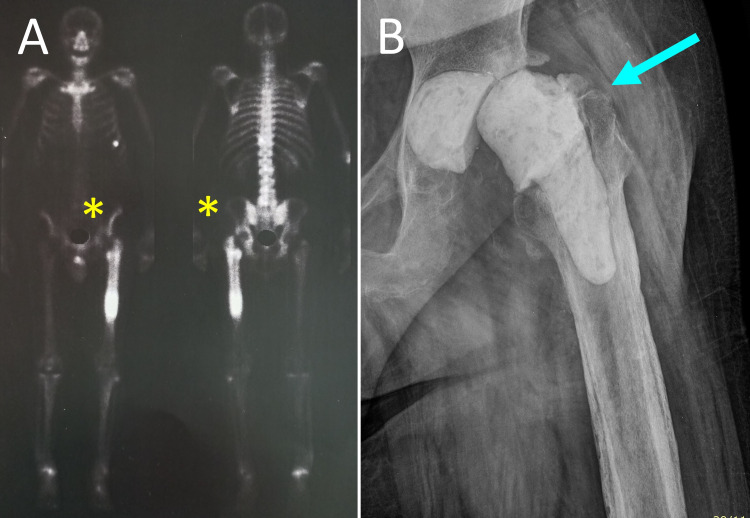
PJI of the left hip of a 65-year-old man with Staphylococcus aureus-infected left THR A. 99m Tc bone scan shows increased radioisotope uptake at the proximal metaphysis and the diaphysis of the left femur (yellow asterisks). B. Anteroposterior radiograph of the left hip after removal of the prosthesis, extensive debridement and implantation of a ball-and-socket ALCS (blue arrow). PJI: prosthetic joint infection, THR: total hip replacement, ALCS: antibiotic-loaded cement spacer

A hand-made ball-and-socket spacer for two-stage hip PJI treatment is shown in Figure 1.

**Figure 2:**
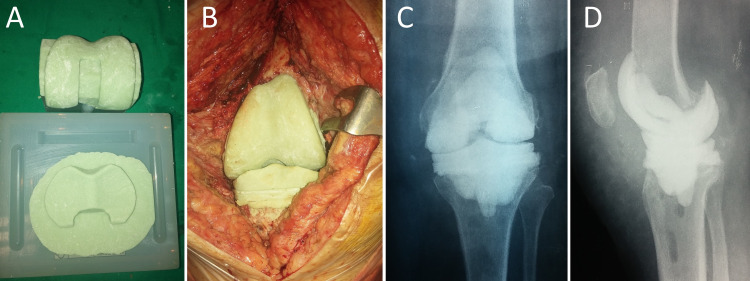
PJI of the left knee of a 75-year-old woman. A. Intraoperative preparation of the articulated ALCS on prefabricated molds. B. Implantation of the articulated ALCS after removal of the infected prostheses and debridement of the left knee. Anteroposterior (C) and lateral (D) radiograph of the left knee after the first-stage revision operation. PJI: prosthetic joint infection, ALCS: antibiotic-loaded cement spacer.

An intraoperatively constructed articulated knee spacer using commercially available molds is shown in Figure 2.

**Figure 3:**
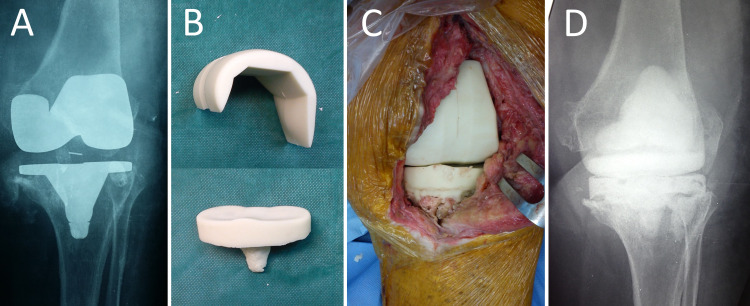
PJI of the left knee of a 69-year-old woman with Staphylococcus epidermidis-infected TKR. A. Preoperative radiograph of the left knee. B. The prefabricated articulated knee ALCS will be implanted after removal of the infected prostheses and extensive debridement and irrigation of the joint space. C. Intraoperative photograph after implantation of the spacer. D. Anteroposterior radiograph of the left knee after spacer implantation. PJI: prosthetic joint infection, ALCS: antibiotic-loaded cement spacer, TKR: total knee replacement.

Knee PJI treated by two-stage revision arthroplasty with interim insertion of a prefabricated ALCS is shown in Figure 3.

The advantage of handmade spacers, compared to commercially available spacers, is their low cost, as they are constructed solely from cement, without the need for special tools. Furthermore, in case of positive preoperative cultures, the antibiotic used for cement impregnation can be selected to target the isolated microbes [9]. Handmade spacers can be loaded with greater amounts of antibiotic than commercial spacers, however, this can lead to suboptimal mechanical properties and increased risk of failure/breakage of the spacer [35,36]. Another disadvantage of handmade spacers is the relatively greater incongruence between the articulating surfaces, which may result in instability due to the lack of tibial post (available in commercial spacers) [9]. The cement-on-cement contact may abrade PMMA particles into the joint and lead to osteolysis [37]. Nevertheless, due to the short-term use of the spacer, particle release does not seem to be a problem in clinical practice [9].

Commercial spacers have superior mechanical properties as far as congruence and strength are concerned. Due to homogenous mixing of cement and antibiotic, the antibiotic is released uniformly into the surrounding tissues [38]. On the other hand, the antibiotic regimen is preloaded and may not target microbes detected on prior diagnostic joint aspiration. Furthermore, the amount of the antibiotic loaded into the spacer is fixed, and cannot be increased, leading to potentially lower effectiveness against microbes [9,39]. However, pre-manufactured spacers have better mechanical properties relative to handmade spacers and present a lower risk for spacer fracture [9].

Static vs Articulating Spacers

Articulating spacers are more popular than static spacers, as they preserve joint mobility between stages of revision. They are associated with decreased muscle atrophy and tend to prevent scar tissue from occupying the joint. Thus, they provide better conditions for second-stage surgery, resulting in superior functional outcomes and a wider postoperative range of motion [1,2,21].

Systematic reviews and meta-analyses comparing static and articulating spacers or the different types of articulating spacers show conflicting results [2,6,21]. Many researchers support the superiority of articulating spacers over the static ones [6,21]. Other studies highlight the increased range of motion of the knee after revision using an articulating spacer, however, they do not report a significantly different final functional outcome compared to static spacers [2].

A recent randomized control trial supports the superiority of the articulating spacers compared to the static ones, in regard to knee range of motion and the Knee Society Score 3.5 years postoperatively [21]. Furthermore, the authors state that the use of static spacers is related to more extensive surgical approaches during the second revision stage and a higher rate of reoperation [21]. Comparison between different types of articulating spacers suggests that they are equally effective to treat PJI, providing similar preoperative conditions for the second-stage implantation and similar functional results of the joint [1,2].

Articulating spacers are indicated in staged revision of most PJI cases, especially when a longer interval (>3 months) between the two revision stages is expected. However, their application can be challenging in the presence of large segmental bone defects, ligamentous instability, or a defective extensor mechanism of the knee, that may ensue after first-stage surgical debridement [1,6,9,21,38]. For this reason, many researchers recommend that the selection of the spacer should be done intraoperatively, after the evaluation of the bone defect and the integrity of the extensor mechanism. Small bone defects and a functional extensor mechanism is an indication for an articulating spacer, while a large defect and/or a defective extensor mechanism is an indication for a static spacer, supplemented by an intramedullary extension (nail, Steinmann pin) to decrease the risk of dislocation and malalignment [38].

Antibiotics for PMMA impregnation

Properties of the Loaded Antibiotics

The antibiotics that are impregnated in the spacer should be heat resistant (up to 82-83⁰C, 12-13 min), so that they are not destroyed during PMMA polymerization [2,40]. They should also be water soluble, to be “washed out” and dispersed into the surrounding tissues after spacer implantation [2]. Antibiotics with small molecular weight are more water soluble [38]. The antibiotics should be chemically stable (neutral) and not react with other molecules contained inside the cement. They should present bactericidal properties, even in low concentrations. The antibiotics should have a low MIC for the targeted microbes. Furthermore, they should not adhere to serum proteins, not provoke allergic reactions and not promote the formation of resistant microbes [11,38].

Aminoglycosides and glycopeptide antibiotics (vancomycin, teicoplanin) are appropriate for PMMA impregnation and are effective against a broad spectrum of microorganisms, commonly isolated in PJI [38]. Antibiotics in powder form (crystalline) are preferred over the liquid form. Antibiotics in liquid form are cheaper but impede polymerization, increase PMMA porosity and decrease the mechanical strength of handmade spacers (strength decreases by 49% and 46% to compression and tension respectively) [41]. In their study, Hsieh et al. found that the use of liquid gentamycin increased the porosity, in contrast to vancomycin that did not [42]. Compressive strength of the cement decreased by 13% when impregnated with vancomycin, 37% when liquid gentamycin was used, and 45% when both antibiotics were added [42]. Therefore, liquid antibiotics should be avoided for cement impregnation, especially in patients, who, for various reasons or comorbidities, may never make it to the second-stage implantation [41].

Antibiotic Selection and Antibiotic Combinations Used for Spacer Preparation

A spacer may contain more than one antibiotics [2]. When the causative bacteria have been identified and are sensitive to antibiotics, a single antibiotic may be used [9]. However, when the causative bacteria are not known, a combination of antibiotics is preferred, to broaden the antimicrobial spectrum (including gram+ and gram- bacteria) of the spacer [2,9]. Antibiotics most commonly used for cement impregnation are tobramycin, gentamycin, vancomycin and cephalosporines [9]. Vancomycin is effective against MRSA, gentamycin against enterobacteria and Pseudomonas aeruginosa, and cefotaxime is effective against gentamycin-resistant bacteria [9]. A popular combination is that of an aminoglycoside (tobramycin or gentamycin) with vancomycin [2,11,38].

The elution of different types of antibiotics from the cement is variable. Using a combination of antibiotics has many advantages over monotherapy. Apart from broadening the antimicrobial spectrum, the combination of an aminoglycoside with vancomycin results in increased release of both antibiotics in vitro, a phenomenon known as passive opportunism [42,43]. The combination of an aminoglycoside with vancomycin is recommended even when the microbe is resistant to aminoglycosides, as high concentrations of aminoglycoside (>3.6g tobramycin and 1g of vancomycin per cement package) promote vancomycin release [2]. The combination of meropenem with vancomycin also results in increased vancomycin release [44].

Except for the commonly used antibiotics (vancomycin, tobramycin, gentamycin), appropriate for cement impregnation are also amikacin, amphotericin B, fluconazole, cefazolin, cefotaxime, cefuroxime, ciprofloxacin, clindamycin, colistin, daptomycin, erythromycin, linezolid, meropenem, piperacillin/tazobactam, teicoplanin/tazobactam, ticarcillin. These antibiotics are reasonable choices when the causative bacteria have been identified preoperatively [2,9,45]. Rifampicin is very effective against the biofilm produced by S. aureus. However, its use for spacer impregnation is contraindicated, as it compromises the mechanical properties of the cement [46]. Tetracycline is effective against both gram+ and gram- bacteria, but is not very heat resistant and may promote bacterial resistance [11,45,47].

Amount of Antibiotic Used for Cement Impregnation

It is well known that the addition of antibiotics impairs the biomechanical properties of the cement in a dose-dependent manner [29,41,42,45]. Therefore, the amount of the antibiotic used for cement impregnation depends on the planned application of the spacer [29,41,42,45].

In relation to orthopaedic infection, the dose of the antibiotic mixed with PMMA may be classified as prophylactic (prevent infection) or therapeutic (treat established infection). Cement used for prosthesis fixation in primary joint replacement is expected to endure long-term full weight-bearing of the patient. This cement should contain a low (prophylactic) dose (1-2g per 40g PMMA) of antibiotic or no antibiotic at all [2,36]. On the contrary, an ALCS is a temporary local carrier of antibiotics. It is used for a short time period, during which partial or no weight-bearing is expected. Thus, an ALCS should be impregnated with high (therapeutic) doses of antibiotics (at least 3.6g of antibiotic per 40g of PMMA) for a two-stage revision for PJI [2,11]. Of note is, that in Europe, cement loaded with prophylactic doses of antibiotics is used for both primary and revision arthroplasties, while in the USA prophylactically impregnated cement is labelled only for revision arthroplasties [11].

Overall, in ALCS’s, the antibiotics should not exceed 10-15% [45] or 20% [29] of the total mass of the cement. Too high antibiotic content of the cement (>8g/40g PMMA) should be avoided, as it affects cement polymerization and impairs its mechanical properties [29,48]. Cements containing 2g of antibiotic per 40g of cement are considered low-dose antibiotic-loaded cements, while cements impregnated with larger amounts of antibiotic are considered as high-dose antibiotic-loaded cements [36,38].

Combination of antibiotics for spacer impregnation varies among authors. In general 1-3g vancomycin combined with 1.2-4.8g of aminoglycoside (gentamycin or tobramycin) is added per 40g of PMMA [2].

Mixing Procedure of the Antibiotic and the Cement

The mixing procedure of the antibiotic and the cement affects antibiotic elution from the spacer. Atmospheric mixing leads to higher porosity, lower spacer strength and increased outer (antibiotic-releasing) surface of the spacer. In hand-made spacers, the antibiotic may not be released uniformly, in contrast to commercial spacers, since the preparation of commercial spacers is standardized and the ingredients are mixed far more homogenously, compared with hand-made spacers [9].

Vacuum mixing decreases cement porosity, and, theoretically, the release of the antibiotic. However, this may not always be the case, as other factors, such as water solubility of the antibiotic or local osmosis, may increase antibiotic elution [49].

For intraoperative ALCS construction, it is recommended that the cement and the antibiotic powder should be mixed homogenously before being added to the liquid monomer [9]. Mixing of the powder and the liquid monomer can be done in atmospheric pressure or in a vacuum. When the mixture starts to solidify, it is molded to the preferred spacer form and is implanted without pressure, to avoid firm adherence to the bone. This facilitates spacer removal during the second-stage surgery [6,9].

Implanting the Spacer

It is common practice to fix spacers to the bone using PMMA. This cement should be loaded with a therapeutic (high) dose of antibiotic (Anagnostakos K, personal communication). After its preparation, the antibiotic-loaded PMMA is first applied on the spacer, while it is in a relatively liquid state, to adhere firmly to the spacer. The PMMA-coated spacer is implanted later into the bone, when the PMMA starts to harden, to allow easy removal during the second-stage surgery [18].

Spacer pharmacokinetics

Factors Affecting Antibiotic Elution from the Spacer

The effectiveness of a spacer as a local carrier of antibiotics depends on its ability to achieve high concentrations of the antibiotic at the site of infection for an appropriate period of time [9,50].

Local antibiotic concentrations should be higher than the MIC of the specific antibiotic against the causative microbe/s [38,51,52]. These concentrations should be maintained at high levels for a prolonged time between the two revision stages in order to eradicate the microbe/s and eliminate the risk for infection recurrence or growth of resistant strains [38,42,52].

Elution of the antibiotic from the spacer is accomplished through a diffusion mechanism. The antibiotic is eluted at first from the cement surface, but its release from the inner cement body continues through a network of channels, cracks, and cavities that are formed by forces created by friction and compression while weight-bearing [9,41,53]. Diffusion rate depends on the dose and type or combination of the loaded antibiotics, on the cement mixing process, on its porosity and the surface morphology of the spacer, which is in contact with the inflamed tissues [9,41,50].

Antibiotic release from PMMA is a surface phenomenon and does not depend on the amount of the cement [54]. It has been reported that the elution of gentamycin is accomplished rapidly through a cement depth of only 100μm from its surface and thereafter is little further leaching [54]. The larger the cement surface in contact with the surrounding tissues, the larger the rate and amount of antibiotic elution from the spacer [9,11,54,55]. An increase in the distance between the cement and the surrounding tissues results in decreased concentration of the antibiotic [55]. The type of tissues around the spacer also affects antibiotic elution from the spacer and antibiotic diffusion into the inflamed tissues. Cortical bone absorbs less antibiotic than cancellous bone and cancellous bone absorbs less antibiotic than the hematoma (dead space) that potentially lies between the spacer and the bone or soft tissues. Therefore, as much contact as possible, between the spacer and the inflamed tissues should be aimed [11,55].

Most studies support that the elution of the antibiotic from the spacer takes place during the first postoperative days (mainly in the first 24 hours), while others support a more timely extended release [24,41,54-56].

There are two phases in the elution of the antibiotic. The first phase, described as burst release phase, occurs minutes or hours after spacer placement and is characterized by very high local antibiotic release [11,24,41]. This rapid release is accomplished through the elution of the antibiotic from the surface of the spacer. The second phase is termed the sustained release phase. It is the continuation of the burst release phase and is characterized by a significantly lower antibiotic concentration, that, however, is maintained for a prolonged period of time [11]. Sustained release results from the penetration of water into the hydrophilic PMMA. This water “washes” the water-soluble antibiotics out of the spacer [11].

Other researchers describe three phases: The exponential phase (during the first postoperative days), the declining phase, and last, the low constant-elution phase [9]. The higher the antibiotic dose that is loaded in the spacer, the higher the antibiotic concentration during the exponential phase [9].

Joint motion and the potential of the tissues to absorb the antibiotic may affect the quantity of the antibiotic released from the spacer [41,53]. This may explain the absence of correlation between antibiotic concentration inside the joint and in the circulation reported in various studies [57].

In Vivo Antibiotic Release

In vitro studies on the release of the antibiotic from the cement do not correlate with in vivo results [2]. An in vitro study by Kelm et al. concludes that the spacer has no antimicrobial effect after 14 days [58]. The important question, regarding a spacer’s efficacy in the treatment of PJI is, whether a spacer can achieve a certain antibiotic concentration in vivo or not [52].

An in vivo study by Downes et al. reports negligible antibiotic levels beyond the 5th postoperative day [56]. Another study by Schurman et al. reported that only 8% of the loaded antibiotic (gentamycin) had been released till the 8th postoperative day, followed by a sustained release of a minimal antibiotic dose [54]. The antibiotic-impregnated beads, on the other hand, are related to a 93% release of the loaded antibiotic [11]. This should always be considered, in case of concomitant placement of a spacer and antibiotic beads, as this could lead to very high antibiotic concentrations during the burst release phase and thus, to a potentially toxic systemic concentration of the antibiotic [59].

Other in vivo studies support that a cement spacer achieves effective antibiotic concentrations throughout the period between the two stages of revision. In a systematic review, the in vivo antibiotic concentrations were measured during the first week after spacer implantation (seven studies), on the day of spacer removal (three studies) or both (one study) [41]. From these studies it is concluded, that, a large increase in the antibiotic concentration is observed on the first day after implantation, followed by a rapid decrease during the next days. Furthermore, the antibiotic concentration at spacer removal (six weeks - four months after implantation), although lower than during the first week, was always higher than the MIC for the causative bacteria [41].

In Vivo Example of Antibiotic Release

The study of Hsieh et al. is an interesting example of spacer pharmacokinetics [24]. The authors conducted an in vivo study of two-stage revision surgery in 46 patients with hip PJI. They used intraoperatively molded ALCS’s, impregnated with high doses of antibiotics (4g vancomycin + 4g aztreonam per 40g of PMMA). The concentration of each antibiotic was measured in the suction drain of the wound on a daily basis during the first postoperative week. The mean vancomycin concentration dropped from 1,538 μg/mL on the 1st postoperative day to 571.9 μg/mL on the 7th day. The mean aztreonam concentration dropped from 1,003.5 μg/mL on the 1st day to 313.6 μg/mL on the 7th day. During the first postoperative week, the patients did not receive any systemic antibiotics. After the first postoperative week, the drains were removed and systemic antibiotics for two weeks were administered [24].

**Figure 4:**
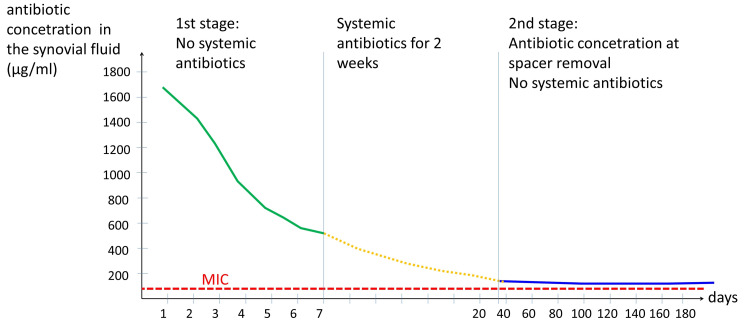
Schematic representation of the concentration of the antibiotic (vancomycin or aztreonam) in the synovial fluid after two-stage revision surgery for hip PJI. The first part of the curve (green line) depicts the concentration of the antibiotic collected daily from the suction drain during the first postoperative week. The second part of the curve (orange dotted line) is hypothetical. It represents the concentration of the antibiotic during systemic antibiotic administration and during drug holiday. The third part of the curve (blue line) represents the concentration of the antibiotic in the synovial fluid, which was obtained by joint aspiration just before the second stage implantation. The red interrupted line is the MIC of the common microorganisms for the antibiotic. Burst release upon ALCS implantation and rapid drop of the concentration of the antibiotic is evident. Sustained release of antibiotic from the ALCS above the MIC of the microorganisms throughout the two-stage revision procedure is observed. Both antibiotics showed similar patterns of elution from the ACLS (Adapted from Hsieh et al. [24]). MIC: Minimum inhibitory concentration, ALCS: Antibiotic-loaded cement spacer, PJI: Prosthetic joint infection.

The change of the concentration of the antibiotics, which were used for cement impregnation, is schematically shown in Figure 4. Both antibiotics showed similar patterns of elution.

The final prosthesis (second-stage procedure) was implanted after a mean interval of 107 days from spacer implantation (first-stage procedure). Prior to surgery, joint fluid was aspirated and the concentrations of vancomycin and aztreonam were measured. The concentration of the antibiotics was well below the immediate postoperative concentration, however, it was always above the MIC of the most common microbes observed in PJIs [24].

The high antibiotic content of the spacer did not lead to a considerable and long-lasting increase of the serum concentrations of vancomycin and aztreonam during the first week after spacer implantation: on the first postoperative day, the mean vancomycin concentration was 0.58μg/mL and the mean aztreonam concentration was 0.46μg/mL. The serum concentrations of the antibiotics dropped to undetectable levels on the 3rd postoperative day. No patient showed systemic adverse effects (renal-, hepatic toxicity, other) [24].

The in vivo spacer pharmacokinetics reported in this study are unique, since antibiotic measurements (synovium, serum) were made during periods the patients did not receive systemic antibiotics. Consequently, the measured amounts of antibiotics originated exclusively from the ALCS [24].

Similar conclusions are made by other studies in regard to the immediate postoperative antibiotic release from ALCS’s [9,11,41,55].

How Long are ALCS’s Effective Antibiotic Carriers?

The interval between the two stages (time of application of the ALCS) should be at least 30 days, so that the antibiotic can penetrate and diffuse into low-quality tissues with poor vascularity, such as scar tissue and sclerotic bone [55]. Prolonged spacer retention may lead to sub-inhibitory concentrations of the antibiotic and promote superinfection with resistant bacteria, however, this risk does not seem to be a problem in the clinical practice and will be discussed later [1,24,41].

Complications of ALCS

ALCS-related complications are quite frequent [1,6,8], affecting 26%-58.5% of patients after staged revision arthroplasty [60,61].

ALCS-related complications may be classified as local or systemic. Articulating ALCS may harm the extensor mechanism of the knee and lead to wound dehiscence [1,2]. Not infrequently, the ALCS may break or small particles may abrade, leading to failure of the final prosthesis [38]. Static ALCS may lead to bone loss [1,2].

**Figure 5:**
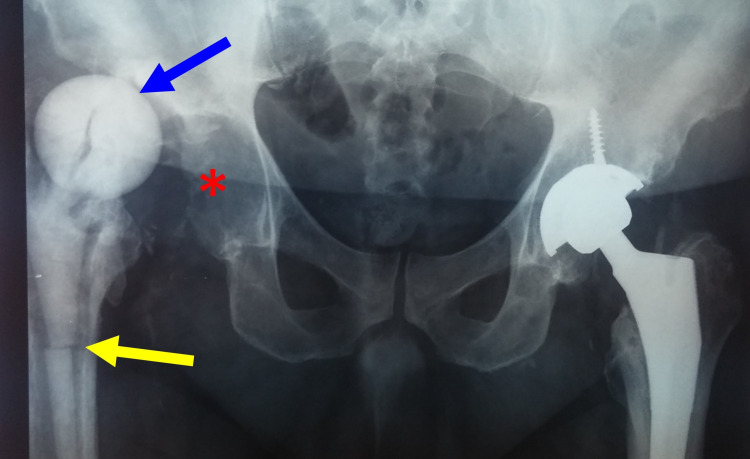
Two-stage revision for PJI after total replacement of the right hip using a prefabricated spacer. Breakage (yellow arrow) and dislocation (blue arrow) of the spacer out of the acetabulum (asterisk). PJI: Prosthetic joint infection.

Hip ALCS may cause a periprosthetic femur fracture. Tilting of the spacer secondary to poor fit into the proximal femur may occur [25] leading to dislocation or subluxation of the ALCS (Figure 5) [1,25, 60-63].

**Figure 6:**
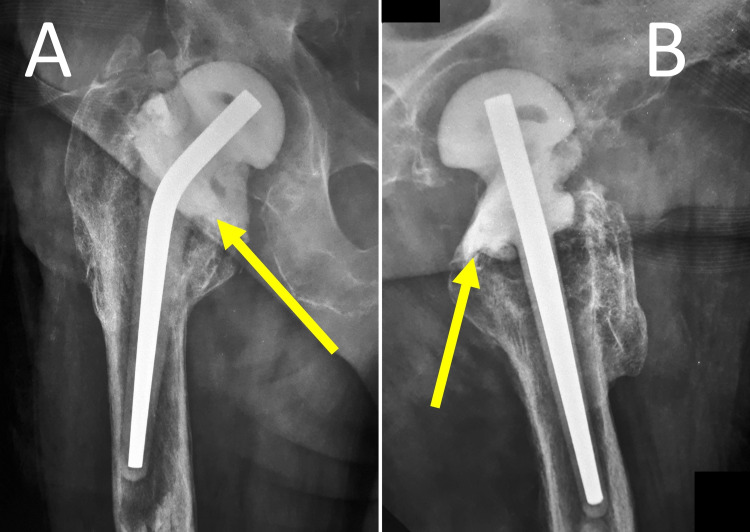
Two-stage revision of PJI after an infected hemiarthroplasty of the right hip using a prefabricated ALCS. Anteroposterior (A) and lateral (B) radiographs of the right hip. High-dose antibiotic-loaded cement (yellow arrows) has been added around the proximal part of the spacer to prevent complications such as migration, rotation and dislocation of the spacer or bone loss of the femur. PJI: Prosthetic joint infection, ALCS: Antibiotic-loaded cement spacer.

To prevent tilting, migration, rotation or dislocation, the proximal part of the spacer should be stabilized into the proximal femur with additional antibiotic-loaded cement (Figure 6) [25].

Systemic complications after ALCS implantation are attributed to the increased release of antibiotics immediately after the first-stage surgery. Such complications are nephrotoxicity, hepatotoxicity, and ototoxicity secondary to gentamycin or allergic reactions and neutropenia secondary to vancomycin [11,38]. Older patients are probably more prone to such complications [38]. Occasionally, removal of the ALCS is mandatory to restore renal function [8,59,64,65]. Increased serum creatinine above 50% from normal has been reported in 20% of patients with tobramycin-impregnated spacer during the first postoperative week after the first-stage surgery [66]. Underlying renal dysfunction predisposes to ALCS-related nephrotoxicity [64-66]. However, other studies support that the high levels of the antibiotics observed immediately after spacer implantation return to normal within 24 hours, therefore systemic side effects are not a potential risk for the patient [9,41,42].

Other authors state that patients which undergo two-stage revision are usually elderly people with co-morbidities, such as chronic renal or hepatic disease. These patients should be monitored closely for systemic adverse effects after spacer implantation, particularly if the spacers are impregnated with high amounts of antibiotics [6], or non-steroid anti-inflammatory medication is administered postoperatively [66].

Local toxic effect after high antibiotic release?

Antibiotics used for cement impregnation may exert local toxicity on cells involved in tissue regeneration, such as osteoblasts, endothelial cells, ﬁbroblasts or skeletal muscle cells [67]. It has been shown that vancomycin exhibits a time-dependent and concentration-dependent inhibition of growth on these cell lines [67]. Other authors tested the potential toxic effect of antibiotics, such as vancomycin, tobramycin and gentamycin on eukaryotic cells in vitro [11]. It has been shown that, on eukaryotic cells, the in vitro toxic concentrations of these antibiotics exceed the maximal therapeutic concentrations observed in vivo [11]. Therefore, in the clinical setting, it is difficult to attribute problems observed after spacer implantation to local toxicity of the released antibiotics [11].

Antibiotic-loaded cement leads to microbe resistance?

The surface of the bone cement favors microbial adherence and colonization, even in the presence of antibiotics [68-70]. The risk of developing microbial resistance may increase when the concentration of the antibiotic drops below therapeutic levels [11,69,71,72].

There are two aspects of this problem:

Regarding cemented primary total joint replacement, it is not clear whether fixation of the implants with antibiotic-loaded cement could increase the risk of microbial resistance and subsequently predispose to future PJI with resistant microbes. Some authors support that prophylactic gentamycin cement impregnation increases the risk for subsequent PJI with gentamycin-resistant microbes [73], while others do not [74,75], nor does prophylactical antibiotic-loaded PMMA of the primary procedure change the pattern of the microbes in case of future PJI [75].

Regarding two-stage revision arthroplasty for PJI, there is controversy, whether the ALCS could lead to the bacterial resistance and secondary superinfection. Ma et al. showed that bacterial 16s rRNA was present on spacers retrieved at the second-stage surgery for PJI [76], indicating that viable bacteria were present on the ALCS during the “drug holiday” before second-stage surgery. The term “bacterial resisters” was used to describe these bacterial strains, which were protected inside a well-formed biofilm [76]. These bacteria could be a source of recurrent infection [76] and may be responsible for the failure of second-stage implantation [77].

Nevertheless, in the clinical setting it is not clear, whether prophylactic or therapeutic application of antibiotic-impregnated cement is a source of bacterial resistance that may predispose to PJI or compromise PJI treatment, respectively [11].

Multiple spacer exchange

One or multiple ALCS exchanges between the first-stage surgery and the final prosthesis insertion are reported in 12-17% of patients with PJI [62,76,78].

Spacer exchange is dictated by mechanical causes or because of persisting infection [62]. Mechanical causes of spacer replacement are the risk of wound or skin compromise secondary to pressure, compression of nerves or vessels and, finally, pain and discomfort from a dislocated spacer. However, if tolerated, spacer exchange may be avoided until final prosthesis implantation [62].

Persisting infection is the most frequent cause of spacer exchange [62]. It is manifested by continuous drainage and local and/or systemic signs of infection. Persisting infection mandates spacer exchange and additional debridement and irrigation of the joint [6,62]. Obese patients or patients with rheumatoid arthritis are more prone to spacer exchange secondary to persisting infection [79]. Patients with spacer exchange have more comorbidities and present more frequently resistant microbes [62]. Failure to complete final prosthesis insertion is observed in 26% of patients with spacer replacement [78], compared with 17.3% of patients without spacer exchange [76].

Patients, who undergo spacer exchange or have a history of multiple surgeries about the joint, usually present multiple comorbidities and have a higher prevalence of PJI due to resistant or difficult to treat microbes [3]. In such cases, salvage procedures should be considered to spare these patients from additional surgical morbidity [62].

Clinical application of ALCS: hip, knee, other joints

ALCS's were introduced to deal with PJI’s after THR and TKR. Increasing total replacement surgery for other major joints, such as the shoulder or the ankle joint, has inevitably led to PJI’s of these joints as well. Thus, two-stage revision with ALCS has been recently described for PJI of the shoulder [80] and the ankle joint [81].

## Conclusions

Prosthetic joint infection is a serious complication after total joint replacement. Two-stage revision arthroplasty using an ALCS is the preferred method of treatment for delayed or late PJI and the last chance of the patient to restore a more or less satisfying quality of life. Suitably shaped antibiotic-loaded cement spacers fill the void, which is left after removal of the infected prosthesis and thorough debridement and irrigation of the joint. Various types of spacers are available, pre-manufactured or handmade, static or articulating. Spacers preserve limb anatomy and some function until the final prosthesis is inserted. Most important, ALCS’s are powerful carriers of antibiotics. Local antibiotic release from an ALCS exceeds by far the antibiotic provided after systemic administration, without the risk of systemic toxic effects. In direct contact with the infected bone and soft tissues, spacers release locally high amounts of antibiotics to eradicate biofilm-protected microbes. Systemic antibiotics “finish the job” to eradicate the remaining microbes. Proper choice of the type and the amount of antibiotics contained in the spacer is important to control infection. ALCS pharmacokinetics indicate that spacers sustain local antibiotic concentration above the MIC of most common microbes between the first- and second-stage surgery. Complications of the spacer, such as migration and breakage, soft tissue pressure, neurovascular compression, pain or persisting infection mandate spacer exchange. Patients with comorbidities, resistant bacteria or multiple previous surgeries at the joint are prone to fail final prosthesis insertion and are candidates for salvage surgery.
